# The Prevalence, Mechanisms, and Clinical Significance of Inferior Vena Cava Compression in Autosomal Dominant Polycystic Kidney Disease: A Multicenter Retrospective Cohort Study Based on TriNetX

**DOI:** 10.3390/medicina62010230

**Published:** 2026-01-22

**Authors:** Ahmad Matarneh, Bayan Matarneh, Abdelrauof Akkari, Sundus Sardar, Omar Salameh, Navin Verma, Nasrollah Ghahramani

**Affiliations:** 1 Division of Nephrology, Department of Medicine, Penn State Health, Milton S. Hershey Medical Center, Hershey, PA 17033, USA; a_akkari_81@yahoo.com (A.A.); ssardar@pennstatehealth.psu.edu (S.S.);; 2 Department of Pediatrics, Children’s Hospital of Michigan, Detroit, MI 48201, USA; drbayanmatarneh@gmail.com; 3 University Health Truman Medical Center, Kansas City, MO 64108, USA

**Keywords:** autosomal dominant polycystic kidney disease (ADPKD), inferior vena cava compression, end-stage renal disease (ESRD), venous thrombosis

## Abstract

*Background and Objectives:* Autosomal dominant polycystic kidney disease (ADPKD) is a leading cause of end-stage renal disease (ESRD). Progressive renal cyst growth in ADPKD can exert mass effects, including compression of the inferior vena cava (IVC), a rare but clinically significant complication with implications for hemodynamic stability and renal outcomes. This study evaluated the prevalence of IVC compression in ADPKD and its impact on progression to ESRD, mortality, and overall survival. We aimed to provide quantitative measures to elucidate its prognostic significance. *Materials and Methods:* Using the TriNetX database, we conducted a retrospective cohort study of 658 ADPKD patients with IVC compression, comparing them to unmatched controls without compression. Outcomes included ESRD incidence, mortality, and survival. Kaplan–Meier curves and hazard ratios (HRs) with 95% confidence intervals (CIs) were used for analysis. *Results*: ESRD Risk: IVC compression was associated with a higher risk of ESRD (77.4% vs. 29.7%, RR: 2.61, 95% CI: 2.49–2.73, *p* < 0.001). Survival Probability: 5-year Survival was significantly reduced in patients with IVC compression (42.6%) compared to controls (61.7%) (HR: 4.00, 95% CI: 3.45–4.63, *p* = 0.002). Mortality: Mortality was higher in the compression group (29.2% vs. 9.1%). Combined Impact: ESRD patients with IVC compression had a lower survival rate (11.9%) than ESRD patients without compression (28.5%) (HR: 5.60, 95% CI: 5.12–6.13, *p* < 0.001). *Conclusions*: IVC compression in ADPKD is associated with significantly worse outcomes, including increased ESRD risk, higher mortality, and reduced survival. These findings underscore the importance of early diagnosis and targeted management strategies.

## 1. Introduction

Autosomal dominant polycystic kidney disease (ADPKD) is the most common hereditary renal disorder, affecting approximately 1 in 400 to 1 in 1000 individuals globally [1]. It is characterized by the progressive development of fluid-filled cysts in the kidneys, which lead to renal enlargement, declining kidney function, and, ultimately, end-stage renal disease (ESRD) in a significant proportion of affected individuals. Beyond the kidneys, ADPKD is a systemic disorder with widespread extrarenal manifestations, including hepatic cysts, intracranial aneurysms, and cardiovascular complications [2]. Despite these well-documented manifestations, the mechanical and vascular consequences of cystic kidney enlargement have received comparatively less attention, particularly their impact on major vascular structures such as the inferior vena cava (IVC) [3].

The IVC, the largest vein in the human body, plays a vital role in venous return to the heart. Its retroperitoneal location makes it susceptible to external compression by the enlarged polycystic kidneys, particularly as renal cysts grow posteriorly [4]. This compression can impair venous outflow, resulting in venous stasis, edema, and, in severe cases, the development of thrombosis. Such complications can significantly exacerbate hemodynamic instability, increase the risk of systemic complications, and accelerate renal functional decline [5]. Despite its potential clinical significance, data on the prevalence, mechanisms, and outcomes of IVC compression in ADPKD remain sparse, leaving an important gap in our understanding of this condition.

Inferior vena cava compression arises from a variety of intra abdominal and retroperitoneal processes. Reported causes include large renal cysts, hepatic cysts, polycystic liver disease, abdominal masses, and retroperitoneal pathology. Case reports describe IVC obstruction due to massive renal cysts and polycystic kidney enlargement [4,5], as well as hepatic cyst expansion and polycystic liver disease producing Budd Chiari like venous outflow obstruction [6]. Additional cases highlight external compression by intra abdominal masses or cyst related hemorrhage [7], indicating that extrinsic IVC narrowing can occur through several benign or malignant mechanisms. Within the context of ADPKD, progressive cystic enlargement of the kidneys and liver represents one of the most common structural causes of significant IVC compression.

Current evidence suggests that patients with advanced ADPKD and significantly enlarged kidneys; high total kidney volume (TKV) are at increased risk of IVC compression, which can lead to venous congestion, lower extremity edema, and thrombosis due to impaired venous return [6]. However, the specific pathophysiological mechanisms, the degree of compression required to cause clinically significant sequelae, and the long-term outcomes for affected individuals have not been comprehensively studied [8]. Addressing these gaps is critical, as timely identification and management of IVC compression could mitigate complications and improve outcomes for patients with ADPKD.

**Figure 1:**
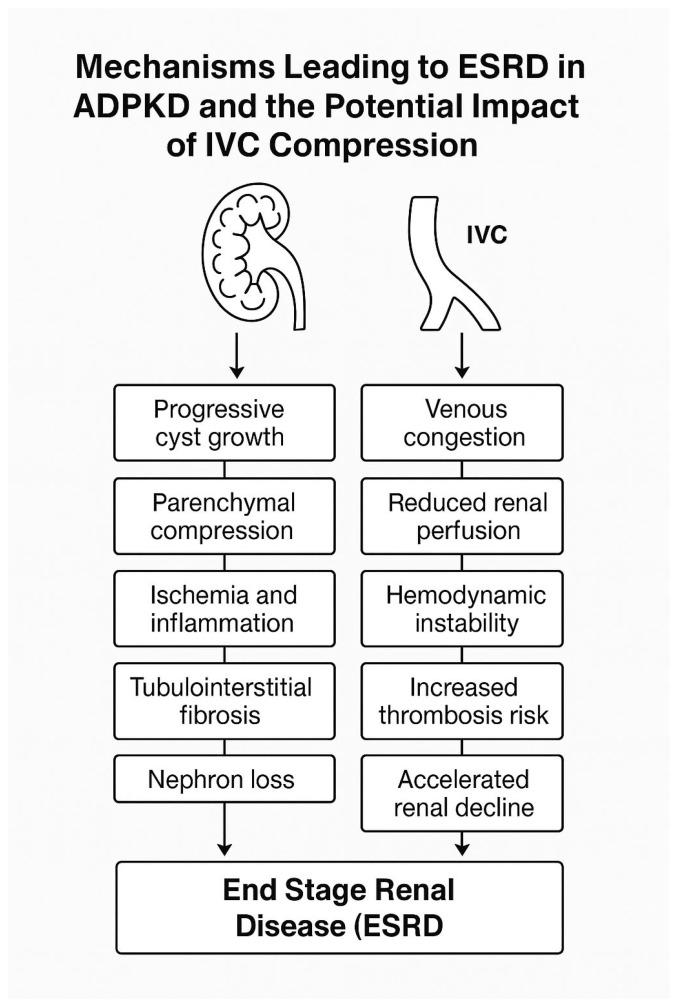
Mechanisms leading to end-stage renal disease in autosomal dominant polycystic kidney disease and the impact of inferior vena cava compression.

End-stage renal disease represents the final common pathway of prolonged nephron loss. In ADPKD, ESRD typically develops as a result of progressive cyst expansion, disruption of normal renal architecture, intrarenal ischemia, and fibrosis. Other kidney diseases can also lead to ESRD, including diabetic kidney disease, hypertensive nephrosclerosis, glomerulonephritides, hereditary nephropathies, and obstructive uropathy [9,10,11]. Within ADPKD, decline in renal function is driven primarily by increasing total kidney volume, which compresses surrounding parenchyma and reduces glomerular filtration efficiency. This chronic pressure related injury accelerates tubular atrophy and interstitial fibrosis, culminating in irreversible renal failure. Given that ESRD is a key clinical outcome in ADPKD, a brief overview of its major etiologies provides context for understanding how structural complications such as inferior vena cava compression may worsen disease trajectory. Mechanisms are summarized in Figure 1.

This study aims to provide a comprehensive assessment of the prevalence, mechanisms, and clinical outcomes of IVC compression in ADPKD. Using data from a large multicenter cohort, we aim to clarify how IVC compression is linked to adverse outcomes, including progression to ESRD, and higher mortality. Through this work, we hope to enhance the understanding of this under-recognized complication and highlight its implications for the management of patients with ADPKD. Although total kidney volume is an important determinant of disease severity in ADPKD, TKV measurements were not available in the TriNetX database. As a result, the study cannot quantify the relationship between kidney size, cyst burden, and inferior vena cava compression, and all mechanistic discussion of TKV should be interpreted conceptually rather than as direct evidence from this cohort.

This figure illustrates how cyst enlargement in autosomal dominant polycystic kidney disease leads to parenchymal distortion, reduced perfusion, inflammation, and tubulointerstitial fibrosis that result in nephron loss. It also shows how inferior vena cava compression adds venous congestion and impaired renal blood flow, which together accelerate progression toward end-stage renal disease.

## 2. Methods

### 2.1. Study Design and Data Source

We conducted a retrospective, multicenter cohort study using data obtained from the TriNetX Research Network. This study included patients with a diagnosis of ADPKD between 1 January 2010, and 31 December 2022, providing a comprehensive dataset spanning over a decade. TriNetX is a global, federated research network that aggregates de identified electronic health record data from participating healthcare organizations for real time cohort discovery and observational research. The platform includes demographic information, diagnoses, procedures, laboratory values, medications, vital signs, and mortality data drawn directly from institutional clinical systems. Its analytic environment allows researchers to perform large scale population based studies using standardized data structures while protecting patient privacy.

### 2.2. Study Population

We used the global health collaborative network on TrinetX. Our Inclusion criteria consisted of adults aged ≥18 years with a confirmed diagnosis of ADPKD based on ICD-10-CM code Q61.2. Diagnostic confirmation was further supported by available clinical or radiological documentation.

Exclusion criteria included patients with incomplete demographic or clinical data, including missing age, sex, or laboratory values relevant to the outcomes.

A total of 658 patients with ADPKD and a structured diagnosis indicating inferior vena cava (IVC) compression were identified. For comparison, we included 31,131 patients with ADPKD who had no recorded evidence of IVC compression. The identification of IVC compression was based on structured diagnostic entries in the EHR; however, central radiologic adjudication or standardized imaging review was not available. The diagnosis of IVC compression in this study was based exclusively on structured electronic health record entries available within the TriNetX platform. Patients were identified using standardized terminology recorded in the diagnosis fields, including terms such as ‘inferior vena cava compression’, ‘compression of IVC’, and ‘vena cava obstruction due to mass effect’. These terms represent clinician-entered diagnoses and do not include centralized radiologic confirmation. Because the TriNetX environment does not provide access to imaging reports or allow adjudication of radiographic studies, misclassification is possible, particularly in cases where the diagnosis may have been entered presumptively or inconsistently across institutions.

### 2.3. Demographics and Clinical Characteristics

Baseline demographic and clinical characteristics were extracted for both groups, including age, sex, race, and ethnicity. Diagnosis of ADPKD was primarily supported by imaging and family history, with genetic testing performed in only 2% of patients. Among those tested, PDGFRA mutations were identified in 10 individuals. Utilization of disease-modifying therapy was low: only 2% of patients with IVC compression had received tolvaptan at any point after their diagnosis. Additionally, 13% of these patients had undergone nephrectomy—either partial, unilateral, or bilateral—as part of disease management. Total kidney volume (TKV) data were not available in the TriNetX dataset, and no imaging derived measurements of kidney size or cyst burden could be included in this analysis.

### 2.4. Baseline Characteristics

Patients in the IVC compression group were slightly older, with a predominant age range of 60–69 years, compared to 50–59 years in the non-compression group. The IVC group had a lower proportion of female patients (45.3% vs. 51.0%) and was more likely to include individuals identifying as Black or African American (24.8% vs. 12.1%) and less likely to include those identifying as White (49.4% vs. 63.2%).

Markers of renal dysfunction were significantly worse in the IVC group, with higher mean serum creatinine (4.91 ± 4.23 vs. 2.59 ± 4.18 mg/dL) and blood urea nitrogen levels (36.55 ± 21.28 vs. 28.25 ± 20.04 mg/dL). Serum phosphate was elevated in the IVC group (4.34 ± 1.76 vs. 3.75 ± 1.29 mg/dL), consistent with more advanced kidney disease. Hemoglobin was lower (11.15 ± 2.77 vs. 12.54 ± 2.45 g/dL), indicating more pronounced anemia of chronic kidney disease.

Markers of inflammation and systemic stress were also elevated in the IVC cohort, including C-reactive protein (64.23 ± 84.54 mg/L), ferritin (900.38 ± 1267.93 ng/mL), and total leukocyte count (14.96 ± 194.08 × 10^9^/L). While data for these markers were incomplete in the non-compression group, the available comparisons suggested a proinflammatory state in IVC patients. In the subset with available echocardiographic data, mean left ventricular ejection fraction (LVEF) remained preserved at 55.6%. Cardiac biomarkers, including BNP and NT-proBNP, were markedly elevated in the IVC group, although available in fewer than half of patients.

Electrolyte abnormalities included modestly lower sodium and calcium levels, and higher potassium, in the IVC group. While body mass index (BMI), blood pressure, and vital signs were inconsistently reported across sites, where available, trends suggested higher systolic blood pressure and heart rate among patients with IVC compression. Baseline characteristics between the two groups are shown in Table 1.

### 2.5. Outcomes

Primary outcomes for this study were defined as follows:

1. Incidence of ESRD: ESRD was determined by the initiation of renal replacement therapy (dialysis or transplantation) or a glomerular filtration rate (GFR) < 15 mL/min/1.73 m^2^.
2. Overall Mortality: All-cause mortality was assessed within the study period.
3. Survival Probabilities: Survival rates were evaluated at the end of the period
4. IVC-Related Complications: This included the incidence of venous thrombosis and associated sequelae.

### 2.6. Statistical Analysis

Analyses were performed using TriNetX’s built-in analytics platform. Categorical variables were summarized as counts and percentages; continuous variables as means ± standard deviations. Group comparisons used chi-square tests for categorical variables and *t*-tests for continuous variables.

Kaplan–Meier survival curves with log-rank tests were used to assess differences in survival. Risk ratios (RRs) and hazard ratios (HRs) with 95% confidence intervals (CIs) were calculated to evaluate associations between IVC compression and outcomes including ESRD, mortality, and thrombosis.

Statistical significance was defined as a two-tailed *p*-value < 0.05. Missing data were excluded from respective analyses. Exact *p* values provided by the TriNetX platform were reported whenever available. For statistics reported as *p* < 0.001, the platform does not provide additional decimal precision. Hazard ratios were generated using the built in Cox proportional hazards function in TriNetX, which adheres to the standard proportional hazards model structure, although formal testing of proportionality is not available within the analytic interface. No matching or propensity score adjustment was performed. Analyses were limited to the built in statistical tools available within the TriNetX platform, which provides cohort level risk ratios and hazard ratios but does not allow user defined multivariable Cox regression or covariate adjustment. As a result, residual confounding due to baseline imbalances cannot be excluded

## 3. Results

### 3.1. Baseline Characteristics

No matching procedures were performed. The two cohorts represent observational groups within the TriNetX network, and the baseline differences observed between them reflect the natural variation in disease severity. These differences should be considered when interpreting outcome comparisons.

### 3.2. Clinical Outcomes

ESRD Risk: The incidence of ESRD was markedly elevated among patients with IVC compression (77.4%) compared to those without compression (29.7%). This translates to a risk ratio (RR) of 2.61 (95% CI: 2.49–2.73, *p* < 0.001), indicating a strong association between IVC compression and renal disease progression.

Survival Analysis: Kaplan–Meier survival curves revealed significantly reduced survival probabilities in patients with IVC compression. At five years, the survival rate was 42.6% in the compression group compared to 61.7% in the control group, corresponding to a hazard ratio (HR) of 4.00 (95% CI: 3.45–4.63, *p* = 0.002).

Mortality: Overall mortality was substantially higher in the compression group (29.2%) compared to the non-compression group (9.1%). This disparity underscores the severe prognostic implications of IVC compression in the ADPKD population.

Venous Thrombosis: The incidence of venous thrombosis was observed in 35% of patients with IVC compression. This complication further exacerbated survival outcomes, with a hazard ratio of 2.84 (95% CI: 2.12–3.54, *p* < 0.001) for thrombotic events compared to patients without compression. Table 2 summarizes the main clinical outcomes in ADPKD patients. 

**Figure 2:**
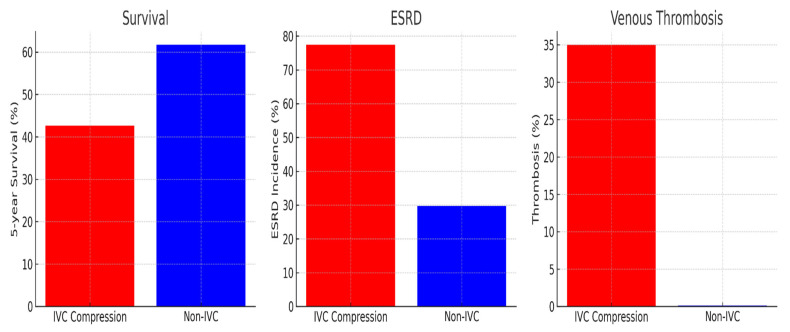
Clinical outcomes in patients with and without inferior vena cava compression.

Combined Impact on ESRD Patients: Among patients who progressed to ESRD, those with IVC compression experienced significantly lower survival rates (11.9%) compared to ESRD patients without compression (28.5%). The hazard ratio for mortality in this subgroup was 5.60 (95% CI: 5.12–6.13, *p* < 0.001), emphasizing the compounded effect of IVC compression and ESRD on patient outcomes. Clinical outcomes are summarized in Figure 2.

This figure compares three major clinical outcomes in autosomal dominant polycystic kidney disease among patients with inferior vena cava compression and those without compression. The left panel shows that five year survival is lower in the compression group. The middle panel shows a higher incidence of end-stage renal disease in patients with compression. The right panel shows a marked increase in venous thrombosis among those with compression compared with those without compression. These findings highlight the potential clinical impact of venous congestion and impaired renal perfusion in the setting of inferior vena cava compression.

## 4. Discussion

This study highlights the significant clinical impact of IVC compression in ADPKD and its association with adverse outcomes, including increased risk of ESRD, reduced survival, and heightened thrombotic complications [9]. IVC compression in ADPKD is not merely incidental but rather an evidence of advanced disease progression, warranting careful assessment and targeted management [12].

IVC compression in ADPKD results from progressive kidney and hepatic cyst expansion, which exerts direct mechanical pressure on the retroperitoneal IVC, leading to venous stasis, congestion, and systemic hemodynamic instability [13]. The impairment of venous return increases venous hypertension, reducing cardiac preload and exacerbating systemic circulation abnormalities. The presence of chronic endothelial dysfunction and venous congestion further predisposes patients to thrombotic events, raising concerns for deep vein thrombosis (DVT) and pulmonary embolism (PE) [14].

Additionally, renal venous congestion due to IVC compression may contribute to glomerular hypertension, intrarenal pressure elevation, and worsening kidney function, potentially accelerating the decline toward ESRD [7].

IVC compression can present with a range of vascular and systemic complications [15], including:

1. Lower extremity edema due to venous congestion and impaired return.
2. Abdominal venous distension and collateral formation, visible on imaging.
3. Exacerbation of renal dysfunction, increasing the risk of rapid kidney disease progression.
4. Thrombophilia, with an increased propensity for DVT, IVC thrombus, and PE, which can lead to significant morbidity and mortality

A particularly concerning complication is IVC thrombosis, which can further worsen venous return, compromise cardiac function, and predispose patients to embolic events [16]. Given these risks, patients with significant IVC compression on imaging should be monitored closely for thrombosis and systemic circulatory instability.

Total kidney volume (TKV) is a well-established marker of disease severity and progression in ADPKD, and increasing TKV is closely associated with IVC compression and its related complications [17]. Studies indicate that TKV and cyst volume increase exponentially in ADPKD, with an average growth rate of 5–6% per year. Severe IVC compression (≥70%) has been observed in approximately 15% of ADPKD patients, while mild compression (≥50% to <70%) is present in another 15% [18].

Patients with TKV exceeding a certain threshold were found to be at significantly higher risk of IVC compression, suggesting a direct correlation between kidney enlargement and vascular involvement [19]. TKV is a well established marker of ADPKD severity in published literature, but TKV values were not available in this dataset. Therefore, references to TKV in this discussion are conceptual and reflect known disease mechanisms rather than findings derived from the present cohort.

These findings suggest that routine monitoring of TKV in ADPKD patients may provide valuable insight into the likelihood of developing IVC compression and its associated complications.

Although genetic testing was available in only a small proportion of patients in this cohort, its importance in ADPKD is increasingly recognized. Molecular diagnosis can confirm disease in individuals with atypical imaging, clarify inheritance patterns, and help stratify patients according to expected disease progression. PKD1 truncating variants are associated with earlier onset of kidney failure and more rapid cyst expansion, whereas non truncating PKD1 variants and PKD2 mutations generally follow a slower course [9,11,20]. Genetic testing therefore provides a framework for identifying individuals who may be more susceptible to complications related to organ enlargement, including extrinsic vascular compression. In this context, genotype data could help refine risk prediction for IVC compression by correlating cyst growth rate, kidney volume trajectory, and extrarenal disease burden with specific pathogenic variants. Incorporating genetic information into future studies may improve phenotypic stratification and support more individualized monitoring strategies. Genetic risk stratification in autosomal dominant polycystic kidney disease relies primarily on identification of pathogenic variants in PKD1 and PKD2, which account for the vast majority of cases. PKD1 truncating mutations are associated with earlier onset of kidney failure, faster cyst expansion, and higher total kidney volume, whereas PKD2 variants generally produce a milder disease course with slower progression. Less common genes linked to atypical or milder cystic phenotypes include GANAB, DNAJB11, and ALG9, which can produce cystic disease with preserved kidney size and later onset of renal dysfunction. Identification of these genes helps refine prognostic expectations and supports individualized monitoring strategies by distinguishing patients at higher risk of accelerated disease progression or extrarenal complications such as vascular compression. Integration of genotype data into future studies of IVC compression may enhance risk prediction by aligning cyst growth kinetics and extrarenal organ enlargement with underlying molecular mechanisms.

Early detection of IVC compression is crucial for risk stratification and management in ADPKD. Magnetic resonance imaging is the preferred modality for evaluating total kidney volume and assessing vascular involvement in autosomal dominant polycystic kidney disease because it provides high contrast resolution and enables visualization of cyst burden and adjacent vascular structures without radiation exposure [4,18]. Computed tomography also offers excellent anatomic detail and is frequently used to characterize the severity of inferior vena cava compression, associated collateral formation, and mass effect from renal or hepatic cysts [5,16]. CT angiography provides additional benefit by allowing detailed assessment of venous flow patterns and identifying thrombotic complications when present [7,21]. Doppler ultrasound is a non invasive bedside modality that can assess venous flow abnormalities and detect thrombotic events, although it has limited sensitivity for identifying extrinsic mechanical compression of the inferior vena cava [7,16].

Given the strong correlation between TKV and IVC compression, routine imaging in high-risk ADPKD patients, such as those with TKV above a critical threshold or rapid kidney growth, may facilitate early intervention and improved patient outcomes.

Since IVC compression is driven by cystic expansion, volume reduction therapies may offer symptomatic relief and reduce vascular complications:

Cyst aspiration and sclerotherapy can temporarily alleviate compression symptoms, though recurrence is common.

Nephrectomy, either unilateral or bilateral, is an option for severe, refractory cases, particularly in patients near ESRD who are candidates for transplantation [20].

Tolvaptan and other cyst growth modulators may slow cyst expansion, although their specific role in mitigating IVC compression remains to be studied further [22].

Screening for IVC compression in ADPKD patients with high TKV or rapid disease progression may provide a valuable opportunity for early intervention. Key areas for future research include:

Defining precise TKV thresholds for increased IVC compression risk to guide clinical decision-making.

Evaluating the effectiveness of volume reduction therapies in reducing IVC compression and associated complications.

Developing standardized screening protocols using imaging to identify high-risk patients before they develop significant complications.

### Limitations

This study has several important limitations. The retrospective design limits the ability to infer causality residual confounding cannot be excluded. Factors such as frailty, socioeconomic conditions, and access to care were not available in the dataset and may have influenced the presence of inferior vena cava compression and subsequent outcomes.

The use of the TriNetX platform introduces variability because it relies on de-identified electronic health record data collected across multiple institutions. Coding accuracy for autosomal dominant polycystic kidney disease, inferior vena cava compression, and end-stage renal disease may vary, and the diagnosis of compression was based on structured clinical entries rather than radiologist-confirmed imaging. A centralized imaging review was not possible.

Because no matching or covariate adjusted Cox regression analysis was performed, baseline imbalances may have influenced the associations observed in this study. The TriNetX platform does not support customized regression modeling or standardized mean difference calculations, which limits the ability to statistically account for these differences. Future work using datasets that allow direct statistical manipulation should incorporate propensity score matching or multivariable adjustment.

Total kidney volume, a key marker of disease severity in autosomal dominant polycystic kidney disease, was not available. This prevented stratification by cyst burden and limited the ability to examine the relationship between kidney size and inferior vena cava compression. Several clinical variables were missing in substantial proportions, including cardiac biomarkers and coagulation parameters, which restricted cardiovascular and thrombotic risk assessment. Hemodynamic consequences of venous compression could not be evaluated because measurements such as central venous pressure are not routinely captured in structured electronic records. A major limitation of this study is the absence of total kidney volume (TKV) data. Because TKV was not available within the TriNetX platform, we could not examine whether kidney size or cyst burden contributed to the likelihood or severity of IVC compression. As a result, no causal or quantitative conclusions about TKV can be drawn from this dataset, and mechanistic interpretations involving TKV should be considered speculative

Only a very small proportion of patients had undergone genetic testing, which prevented meaningful analysis of genotype-phenotype associations. The lack of a uniform definition of clinically significant compression across institutions may have introduced heterogeneity in exposure classification, and the severity or chronicity of compression could not be assessed.

Information on therapies that may influence outcomes was incomplete, including use of tolvaptan, anticoagulation, nephrectomy, cyst reduction procedures, and vascular interventions. As a result, treatment effects could not be evaluated.

Because the dataset reflects practice patterns across diverse health systems, differences in imaging availability, diagnostic thresholds, and dialysis initiation criteria may have contributed to variability. Generalizability outside the United States may be limited. In addition, the timing of inferior vena cava compression relative to end-stage renal disease or mortality could not be established, creating potential temporal ambiguity and the possibility of immortal time bias.

Finally, the diagnosis of inferior vena cava compression likely depended on cross sectional imaging performed more often in symptomatic or advanced cases, which may have contributed to selection bias and may have strengthened the observed association between compression and adverse outcomes.

## 5. Conclusions

IVC compression in ADPKD is an under-recognized but clinically significant complication that is strongly associated with accelerated kidney disease progression, increased risk of thrombosis, higher mortality, and reduced survival. Early recognition with CT or MRI and multidisciplinary management may help mitigate risks, although robust evidence for targeted interventions is still lacking. These results should be interpreted with caution given the retrospective, coding-based nature of the study and the absence of total kidney volume data. Prospective, imaging-based studies are needed to validate these findings and to define evidence-based strategies for screening and management.

## Figures and Tables

**Table 1:** Baseline Characteristics of ADPKD Patients with and without IVC Compression.

Variable	IVC Compression Group	Non-IVC Group
Age (mean category)	60–69	50–59
Sex (Female)	45.3%	51.0%
Race (White)	49.4%	63.2%
Creatinine (mg/dL)	4.91 ± 4.23	2.59 ± 4.18
Urea Nitrogen (mg/dL)	36.55 ± 21.28	28.25 ± 20.04
Phosphate (mg/dL)	4.34 ± 1.76	3.75 ± 1.29
Hemoglobin (g/dL)	11.15 ± 2.77	12.54 ± 2.45
Albumin (g/dL)	3.67 ± 0.89	N/A
C-Reactive Protein (mg/L)	64.23 ± 84.54	N/A
Ferritin (ng/mL)	900.38 ± 1267.93	N/A
Leukocytes (10^9^/L)	14.96 ± 194.08	8.99 ± 85.76
Brain Natriuretic Peptide (BNP) (pg/mL)	2512.91 ± 8960.80	N/A
NT-proBNP (pg/mL)	13,734.80 ± 18,239.80	N/A
Left Ventricle Ejection Fraction (%)	55.58 ± 12.57	N/A
Platelets (10^9^/L)	183.18 ± 87.52	224.44 ± 80.77
Sodium (mmol/L)	137.83 ± 3.80	138.92 ± 3.21
Potassium (mmol/L)	4.51 ± 0.72	4.28 ± 0.57
Bicarbonate (mmol/L)	24.69 ± 4.41	24.72 ± 3.73
Calcium (mg/dL)	9.05 ± 0.95	9.29 ± 0.69
Magnesium (mg/dL)	2.02 ± 0.45	1.94 ± 0.47
BMI (kg/m^2^)	27.56 ± 6.78	N/A
Systolic Blood Pressure (mmHg)	127.88 ± 29.23	N/A
Heart Rate (bpm)	80.52 ± 17.79	N/A

N/A: Results were not available due to limitations of TrinEtX.

**Table 2:** Clinical Outcomes in ADPKD Patients with and without IVC Compression.

Clinical Outcome	IVC Compression (%)	No Compression (%)	RR or HR (95% CI)	*p*-Value
ESRD Incidence	77.4%	29.7%	RR: 2.61 (2.49–2.73)	<0.001
5-Year Survival Rate	42.6%	61.7%	HR: 4.00 (3.45–4.63)	0.002
Overall Mortality	29.2%	9.1%	RR: 3.22	—
Venous Thrombosis Incidence	35.0%	0.12%	HR: 2.84 (2.12–3.54)	<0.001
Survival among ESRD Patients	11.9%	28.5%	HR: 5.60 (5.12–6.13)	<0.001

## Data Availability

The data that support the findings of this study are available from the TriNetX Research Network but restrictions apply to the availability of these data, which were used under license for the current study and are not publicly available. Data may be available from the authors upon reasonable request and with permission from TriNetX.
